# The Role of Local Backrub Motions in Evolved and Designed Mutations

**DOI:** 10.1371/journal.pcbi.1002629

**Published:** 2012-08-02

**Authors:** Daniel A. Keedy, Ivelin Georgiev, Edward B. Triplett, Bruce R. Donald, David C. Richardson, Jane S. Richardson

**Affiliations:** 1 Department of Biochemistry, Duke University Medical Center, Durham, North Carolina, United States of America; 2 Department of Computer Science, Duke University, Durham, North Carolina, United States of America; Bar Ilan University, Israel

## Abstract

Amino acid substitutions in protein structures often require subtle backbone adjustments that are difficult to model in atomic detail. An improved ability to predict realistic backbone changes in response to engineered mutations would be of great utility for the blossoming field of rational protein design. One model that has recently grown in acceptance is the backrub motion, a low-energy dipeptide rotation with single-peptide counter-rotations, that is coupled to dynamic two-state sidechain rotamer jumps, as evidenced by alternate conformations in very high-resolution crystal structures. It has been speculated that backrubs may facilitate sequence changes equally well as rotamer changes. However, backrub-induced shifts and experimental uncertainty are of similar magnitude for backbone atoms in even high-resolution structures, so comparison of wildtype-vs.-mutant crystal structure pairs is not sufficient to directly link backrubs to mutations. In this study, we use two alternative approaches that bypass this limitation. First, we use a quality-filtered structure database to aggregate many examples for precisely defined motifs with single amino acid differences, and find that the effectively amplified backbone differences closely resemble backrubs. Second, we directly apply a provably-accurate, backrub-enabled protein design algorithm to idealized versions of these motifs, and discover that the lowest-energy computed models match the average-coordinate experimental structures. These results support the hypothesis that backrubs participate in natural protein evolution and validate their continued use for design of synthetic proteins.

## Author Summary

Protein design has the potential to generate useful molecules for medicine and chemistry, including sensors, drugs, and catalysts for arbitrary reactions. When protein design is carried out starting from an experimentally determined structure, as is often the case, one important aspect to consider is backbone flexibility, because in response to a mutation the backbone often must shift slightly to reconcile the new sidechain with its environment. In principle, one may model the backbone in many ways, but not all are physically realistic or experimentally validated. Here we study the "backrub" motion, which has been previously documented in atomic detail, but only for sidechain movements within single structures. By a twopronged approach involving both structural bioinformatics and computation with a principled design algorithm, we demonstrate that backrubs are sufficient to explain the backbone differences between mutation-related sets of very precisely defined motifs from the protein structure database. Our findings illustrate that backrubs are useful for describing evolutionary sequence change and, by extension, suggest that they are also appropriate for rational protein design calculations.

## Introduction

Proteins routinely incorporate amino acid changes over evolutionary time by adapting their conformation to the new sidechain. However, it remains a difficult task to predict such a conformational response, especially when subtle backbone adjustments are involved. This issue is of central importance to the burgeoning field of computational protein design, which has recently enjoyed a string of exciting developments [1]–[4].

A number of descriptions of backbone motion have been implemented for the purposes of protein design in the past, each with its own set of advantages and disadvantages. Anticorrelated “crankshaft” adjustments of the ψ(i−1) and φ(i) torsions [5] are evident from order parameters derived from molecular dynamics (MD) simulations, but unrealistically distort the ends of the peptide if employed in isolation. Helical parameters [6] and normal mode analysis [7] enable efficient exploration of conformational space near the starting model, but are only useful for a small subset of protein architectures: respectively, coiled-coils and structures for which a small number of motional modes dominates conformational diversity. Peptide fragments [8] implicitly reflect local protein energetics because they are extracted from experimental structures, but can be computationally inefficient because most random fragment insertion attempts are incompatible with a given local structural context and will therefore be rejected. This being the case, it may be prudent to let nature inform our notion of backbone motion by using a move set based on empirical observations, which may encode aspects of protein energetics and sidechain/backbone coupling that are difficult to handle explicitly.

**Figure 1:**
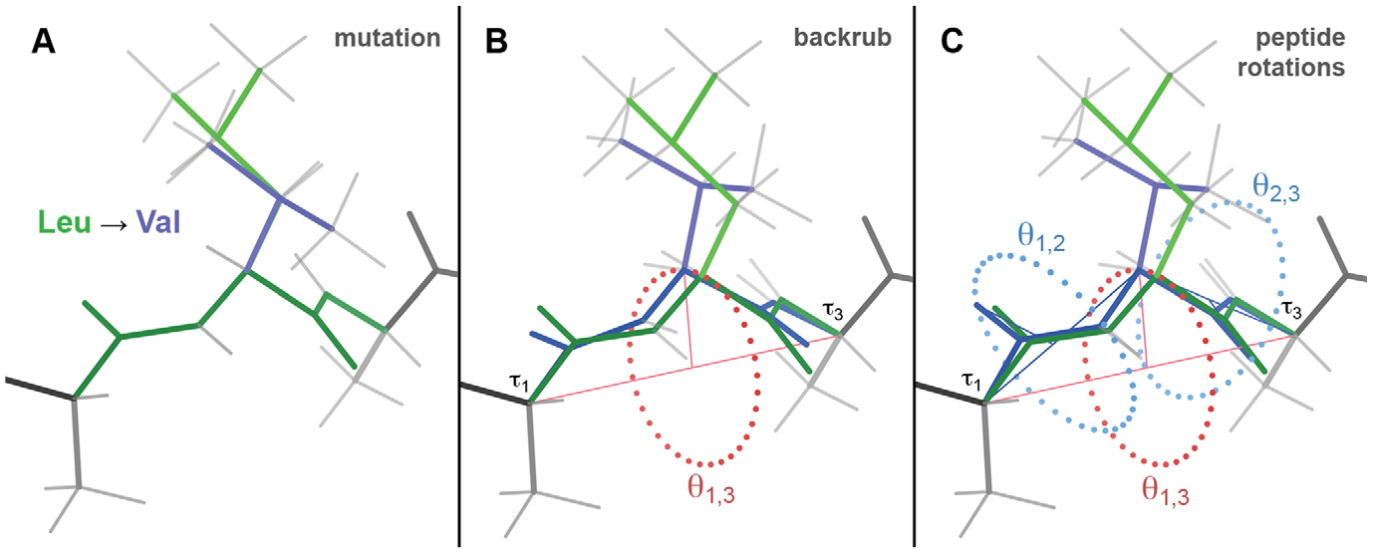
The backrub move for mutation-coupled local protein backbone adjustment. (**A**) A theoretical mutation in ideal β-sheet, from Leu in the **mt** rotamer (green) to Val in the **m** rotamer (blue) [43], changes the interactions of the sidechain with its surroundings. Hydrogen atoms are shown in gray. (**B**) The primary backrub rotation angle θ_1,3_ (red dotted circle) rotates the dipeptide of interest around the C_α1_–C_α3_ axis (red line). As a result, the sidechain of residue 2 (the central residue) swings in a hinge-like manner. In the theoretical example shown, the space occupied by the new Val sidechain is now more similar to the space originally occupied by the Leu sidechain. (**C**) The secondary peptide rotation angles θ_1,2_ and θ_2,3_ (blue dotted circles) counter-rotate the individual peptides around the C_α1_–C_α2_ and C_α2_–C_α3_ axes (blue lines) to alleviate any strain introduced into the flanking τ_1_ and τ_3_ bond angles, respectively, and to restore H-bonding of the two peptides' amides and carbonyls, if necessary. The rotation angles, including the primary backrub angle θ_1,3_, define a motion, not a structure, and thus are meaningful only in reference to a pair of conformations (e.g. before vs. after or mutant vs. wildtype).

One such model is the backrub (Figure 1), a highly localized backbone motion tightly coupled to sidechain rotamer jumps, initially characterized by examining alternate conformations in ultra-high-resolution crystal structures [9]. A simple geometrical model of the backrub consists of a small (<15°) rotation of a dipeptide about the axis between the first and third C_α_ atoms. Resulting strain in the N-C_α_-C bond angle τ of all three residues may be partially alleviated and backbone H-bonding maintained with small counter-rotations of the two individual peptides. Note that this C_α_ formulation is a simplified but very close approximation of the real molecular mechanism, which probably involves a computationally unwieldy set of small shifts in 6–10 backbone torsion angles, as discussed in [9]. Backrubs were seen for 3% of the total residues in that previous study, and for 2/3 of the alternate conformations with a change in C_β_ position – far exceeding the next most common shifts, which are either peptide flips or local shear in a turn of helix.

Several studies have successfully used the backrub approach to expand the search space of protein design efforts and improve agreement between computed sidechain dynamics and nuclear magnetic resonance (NMR) measurements [10]–[14]. Recent work has shown that computational design of backbone structures generated by backrub sampling can recapitulate much of the sequence diversity found in the natural ubiquitin protein subfamily [15] and by phage display experiments [16]. However, the backrub has only been empirically demonstrated to accompany dynamic rotamer changes, not actual changes in amino acid identity. Importantly, no direct experimental evidence has been presented to support the assumption implicit in these studies that a dynamic, low-energy motion on the pico-to-nanosecond timescale is relevant on an evolutionary timescale. The contribution of this current study is to address in atomic detail the specific mechanisms by which backrubs accommodate amino acid changes during processes like subfamily evolution. We use a data set of 5200 high-resolution, high-quality crystal structures to examine differences in local backbone conformation between well-defined motif populations related by a single amino acid difference, and find that the backrub motion explains the majority of the mainchain movement. Furthermore, we demonstrate that a provably-accurate flexible-backbone design algorithm allowing backrubs at those positions, in conjunction with a common molecular mechanics force field, accurately recapitulates such mutation-coupled backbone changes. These findings validate inclusion of the empirically observed backrub motion as part of the repertoire of “moves” for protein design and other modeling efforts.

## Results

### Backrubs of α-Helix N-caps

The N-cap or C-cap position of a helix is defined as the residue half-in and half-out of the helix: the peptide on one side of the cap makes standard helical backbone interactions, while the peptide on the other side has quite non-helical position and interactions [17]. α-helix N-cap residues can make several types of interactions that stabilize or specify the structural transition from loop into α-helix, the most common and dominant of which is a sidechain-mainchain hydrogen-bond to the *i*+3 amide [17]–[19]. The N-cap H-bond enhances protein stability by compensating for the loss of a mainchain H-bond at the helix start relative to the middle of a helix. Note that the sidechain cannot reach this H-bonding position if the residue has helical φ,ψ, so this interaction also specifies the exact helix start position and the direction from which the backbone can enter [20]. Asn, Asp, Ser, and Thr are especially favored at N-caps because their sidechains have the proper chemical character and shape to mimic the helical backbone interactions (which Gln and Glu are too long to do).

Notably, Asn/Asp sidechains are longer than Ser/Thr sidechains by one covalent bond, yet their H-bond distances (N-cap sidechain O to *i*+3 amide H) are only slightly shorter (2.01±0.18 vs. 2.17±0.18 Å) based on a survey of all N-caps with *i*+3 H-bonds in the Top5200 database (described below and in Methods). This means the backbone must slightly adjust to maintain similar H-bond geometry in both cases.

With this motivation, we wished to confirm the appropriateness of the backrub model for this case of mutational rather than rotamer change. However, backbone coordinate shifts due to backrubs are very small – on the order of the coordinate differences between crystal structures of the same protein [21], [22], thus obscuring differences between genuine shifts and experimental noise. The initial description of the backrub bypassed this problem by comparing alternate conformations within single structures [9]. Our approach here, in contrast, was to use the collective weight of many examples to ensure that observed local conformational differences were in fact genuine.

#### N-cap crystal structures

**Figure 2:**
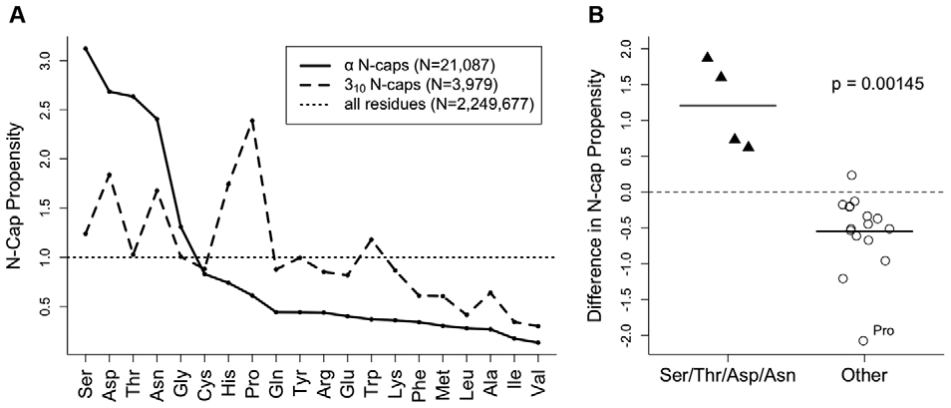
N-cap propensities vary by amino acid type. (**A**) The 20 amino acid types are shown ranked according to their α-helix N-cap propensity (solid line), defined as the fraction of α-helix N-cap residues of the given amino acid type, divided by the fraction of general case residues of that amino acid type (dotted line). The correlation with the analogously defined 3_10_-helix N-cap propensity (dashed line) is surprisingly weak, except for both slightly disfavoring hydrophobics. For example, Ser/Asp/Thr/Asn are the most common N-caps for α-helix but are not especially favored as N-caps for 3_10_-helix. Some other hydrophobic amino acids like Ala/Ile/Val are uncommon as either type of N-cap. (**B**) The canonical α-helix N-caps Ser/Thr/Asp/Asn (triangles) are grouped separately from the other 16 amino acid types (circles); the two groups are compared based on the difference between α-helix N-cap propensity and 3_10_-helix propensity. The horizontal dotted line at 0.0 indicates neither an increase nor a decrease in preference for α-helix N-caps instead of 3_10_-helix N-caps. A one-tailed Mann-Whitney test shows with 95% confidence (p-value = 0.00145<α = 0.05) that Ser/Thr/Asp/Asn are statistically unique in terms of their specificity for α-helix N-caps.

First, we needed to determine which subset of helix N-cap conformations would be likely to undergo backrubs and thus merited further examination. To do so, we compared amino acid preferences for α-helix N-caps and 3_10_-helix N-caps relative to general-case protein structure, using a non-redundant, quality-filtered set of structures, the Top5200 database (see Methods). Asn/Asp/Ser/Thr were indeed found to be strongly preferred (by factors of 2.5–3) at α-helix N-caps relative to protein structure in general (Figure 2). Gly is next most common, but cannot form or be influenced by an N-cap H-bond. Pro is the outstandingly preferred residue at 3_10_ N-caps (Figure 2), while for α-helix Pro is disfavored at the N-cap but occurs preferentially at N-cap *i*+1. These comparative preferences are detailed further in Text S1, with the conclusion that helix N-termini might well respond to a deletion in the preceding loop by transforming from a classic α-helix with Pro at N-cap *i*+1 to a tighter-wound 3_10_-helix with the Pro as N-cap (Figure S1 in Text S1). As an additional factor in our choice of examples, a 3_10_-helix N-cap would have an *i*+2 sidechain-backbone H-bond rather than the *i*+3 common at α-helix N-caps, introducing another complicating issue. Therefore, we decided to focus on Asn/Asp vs. Ser/Thr α-helix N-caps with *i*+3 sidechain-backbone H-bonds in this study.

With this specific motif in mind, we sought to obtain a preponderance of evidence for a backrub relationship in the form of numerous examples. Therefore, we performed a stringent structural motif search of the Top5200, resulting in identification of 429 Asn/Asp N-caps and a matching sample choice of 500 Ser/Thr N-caps (out of 3208; see Methods).

**Figure 3:**
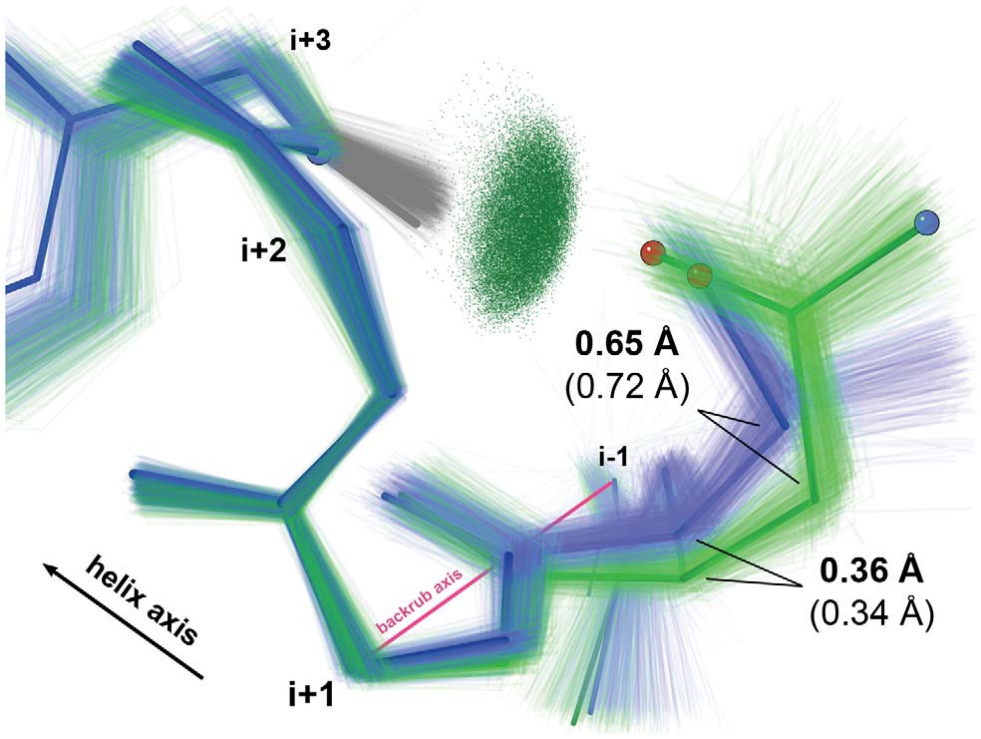
Backrubs at α-helix N-caps. Crystal structure ensembles for Asn/Asp (light green) vs. Ser/Thr (light blue) at the N-cap position are related by a backrub. Lowest-energy BRDEE conformations for the N-terminus of an ideal α-helix (see Methods) with Asn (dark green) vs. Ser (dark blue) have a closely similar relationship. C_α_ and C_β_ displacements between Asn/Asp and Ser/Thr for both average crystal structures (lighter, in parentheses) and low-energy BRDEE conformations (darker) evoke a hinge-like backrub operation. Ensemble *i*+3 sidechain-mainchain N-cap H-bonds are illustrated with “pillows” of green all-atom contact dots [44].

The backbone conformations differ consistently: the longer Asn/Asp sidechains rotate the first turn's backbone away from residue *i*+3, while the shorter Ser/Thr sidechains pull the first turn's backbone toward *i*+3 in order to form the N-cap H-bond successfully (Figure 3, Table 1). The Dataset S1 kinemage shows the full sets of Asn/Asp and Ser/Thr N-caps and animates between them. When average Asn/Asp and Ser/Thr structures are superimposed using the C_α_s surrounding the N-cap in the first turn (N-cap *i*−1 and *i*+1 to *i*+3), all C_α_s match well except the N-cap C_α_ itself (Table 1). The conformational difference at the N-cap position is well modeled by a backrub rotation of about 11°, similar to shifts typical of rotamer-change backrubs. Furthermore, for both N/D and S/T, the C_β_ deviations [23] and the C_α_-C_β_-C_γ_ bond angle distribution at N-caps are close to the general case distribution (Figure S2 in Text S1; further details in Text S1). This means the observed C_β_ shifts and further leveraged sidechain shifts can be attributed primarily to backbone motion rather than altered covalent sidechain geometry.

**Table 1:** Backrub changes for Ser/Thr vs. Asn/Asp α-Helix N-caps.

Structures	Average Crystal	BRDEE Ideal N-cap
ΔC_α_ *i*−1 (Å)	0.03	—
ΔC_α_ *i* (Å)	0.34	0.36
ΔC_α_ *i*+1 (Å)	0.03	—
ΔC_α_ *i*+2 (Å)	0.03	—
ΔC_α_ *i*+3 (Å)	0.04	—
ΔC_β_ *i* (Å)	0.72	0.65
Backrub (°)	−11	−12
Δτ *i*−1 (°)	−0.4	−4.9
Δτ *i*+1 (°)	+2.8	+6.9
S/T HB (Å)	2.18±0.15	2.35
N/D HB (Å)	1.92±0.12	2.00

Distances are after superposition into the same reference frame using 4 C_α_s (N-cap *i*−1 and *i*+1 to *i*+3).

Distances for BRDEE for atoms at or beyond Cα *i*±1 are not shown (marked as “—”) because, by construction, those atoms are not moved by BRDEE.

The signs of the backrub rotation angles and Δτ values are in terms of Ser/Thr→Asn/Asp.

For average crystal structures, average sidechains (based on average C_β_ positions and χ dihedral angles) were added in KiNG. The τ value used for each Δτ is an average across the crystal structure ensemble; this was preferable to measuring τ values directly from the average structures because the average coordinates before the *i*−1 C_α_ were unreliable due to variability within the crystal structure ensemble.

For input to BRDEE, ideal sidechains were added in KiNG to ideal helices. The τ value used for each Δτ is taken directly from the lowest-energy computed structure.

S/T HB and N/D HB are Ser/Thr and Asn/Asp H-bond lengths from the *i* sidechain O to the *i*+3 mainchain H. For crystal structures, an average ± standard deviation across the set of examples in this data set is given. For BRDEE, the value is taken directly from the final model.

The median Asn N-cap sidechain built onto the average Ser N-cap backbone results in a steric clash in which the van der Waals radii of the N-cap sidechain O_δ_1 and the *i*+3 backbone amide H overlap by >0.45 Å. No χ dihedral adjustments can alleviate this clash without abolishing the N-cap sidechain-mainchain H-bond or introducing clashes to other nearby atoms whose positions are fixed for this motif (e.g. the *i*+4 C_β_). Therefore, the backrub away from the first turn by Asn N-caps is indeed forced by steric repulsion.

We also examined two control cases with similar backbone geometry but different sidechain-mainchain interactions. First, we identified 538 α-helix N-caps with any amino acid type except Asn/Asp/Ser/Thr, in which case the *i*+3 sidechain-backbone H-bond is absent. Second, we chose 500 examples of mid-α-helix structure flanked by at least four α-helical residues in both directions, in which case the *i*+3 sidechain-backbone H-bond of an N-cap is satisfied by a usual *i*+4 backbone-backbone α-helical H-bond. The Dataset S1 kinemage shows that the average C_α_ atoms for both control categories are in between the average C_α_ atoms for the Asn/Asp and Ser/Thr categories at the N-cap (or structurally equivalent) residue. This confirms that Asn/Asp and Ser/Thr N-caps are backrub-mediated excursions in opposite directions from equilibrium N-cap/helix structure.

#### N-cap BRDEE computation

We next wished to test whether a simple energy function based on molecular-mechanics terms from Amber [24] and a solvation term from EEF1 [25] would recapitulate these empirically observed changes, given the chance to access them via a backrub. This question is important for solidifying the connection between natural protein evolution and computational protein design. We therefore turned to Backrub Dead-End Elimination (BRDEE) [10], a protein design algorithm that incorporates backrubs in a provably-accurate dead-end elimination framework. BRDEE is freely available as part of the Open Source Protein Redesign for You (OSPREY) [26] suite of protein design software.

As input to the algorithm, we prepared two versions of an idealized helix N-cap motif (see Methods), one with a short sidechain (Ser) and another with a long sidechain (Asn). We then used BRDEE to compute the lowest-energy model for each template, allowing backrubs and rotamer changes at the N-cap as well as small *i*+3 peptide rotations (see Methods).

The lowest-energy Ser N-cap shifted “forward” whereas the lowest-energy Asn N-cap shifted “backward” in order to establish comparable hydrogen bonds (Table 1) in a manner remarkably similar to the empirically observed structures (Figure 3). In particular, the computed and observed C_α_ and C_β_ shifts and inferred backrub angles are of similar magnitude and directionality (Table 1).

The changes in flanking N-C_α_-C angles (τ) for the BRDEE lowest-energy conformations recapitulate the changes for the crystal structures in terms of directionality: Δτ<0 for *i*−1 and Δτ>0 for *i*+1 from Ser/Thr to Asn/Asp (Table 1). The magnitudes are somewhat exaggerated for the computed models, but this is expected because the backrub paradigm somewhat unrealistically redirects strain to these angles as a simplification. That said, we find some evidence of actual systematic bond angle adjustment in the Δτ of almost 3° between the two crystal structure populations (Table 1). There is precedent for context-dependent τ variation with φ,ψ [27], [28]; here the variation appears to be dependent on the presence of a local motif where sidechain-mainchain H-bonding may energetically counteract the bond angle strain. At any rate, actual structures almost certainly undergo more complicated backbone changes that propagate to the *i*±2 residues and alleviate *i*±1 τ angles. However, those additional changes are quite small, and thus the backrub remains an efficient yet remarkably accurate model for conformational changes at N-caps and elsewhere.

Additional comparison and contrast of the computed models and experimental structures can be found in Text S1.

The BRDEE results recapitulate the average crystal structures, confirming the hypothesis that Ser/Thr→Asn/Asp mutations at N-caps are well modeled by a backrub relationship. More generally, this implies that the backrub may reasonably accompany mutations during natural evolution or *in silico* protein engineering.

### Backrubs of Aromatics in Antiparallel β-Sheet

Aromatic residues often pair with glycine in antiparallel β-sheet by adopting rotamers with χ_1_≈+60°, which places the aromatic ring directly over a Gly on the adjacent strand across a narrow pair of backbone H-bonds [29]. Aromatic-glycine pairings in antiparallel β-sheet have been demonstrated to yield a synergistic thermodynamic benefit [30]. If the opposite residue is changed to anything other than Gly, a sidechain including at least a C_β_ atom is now present, which would sterically clash with the aromatic in its original conformation. However, the “plus χ_1_” aromatic rotamer will still be compatible with some rotamers of the opposite sidechain, provided that the aromatic may shift slightly to re-optimize packing of its ring against the opposite residue's C_β_ hydrogens. Here we investigate whether backrubs enable this relaxation by excursions in both directions from a “neutral” β-sheet conformation. The leverage provided by such backbone motions could lean the aromatic residue forward/backward to maintain close inter-strand contact when the identity of the opposite residue is changed to/from Gly.

#### β aromatic crystal structures

A stringent structural motif search, similar to that described for N-caps above, identified 321 Phe/Tyr residues with “plus” χ_1_ rotamers in antiparallel β-sheet (see Methods). Aromatics are about three-fold as common in antiparallel vs. parallel β-sheet, and are about twice as likely to adopt a plus χ_1_ rotamer when they do occur in antiparallel vs. parallel β-sheet (data from Top5200), so we focused on antiparallel β-sheet in this study.

**Figure 4:**
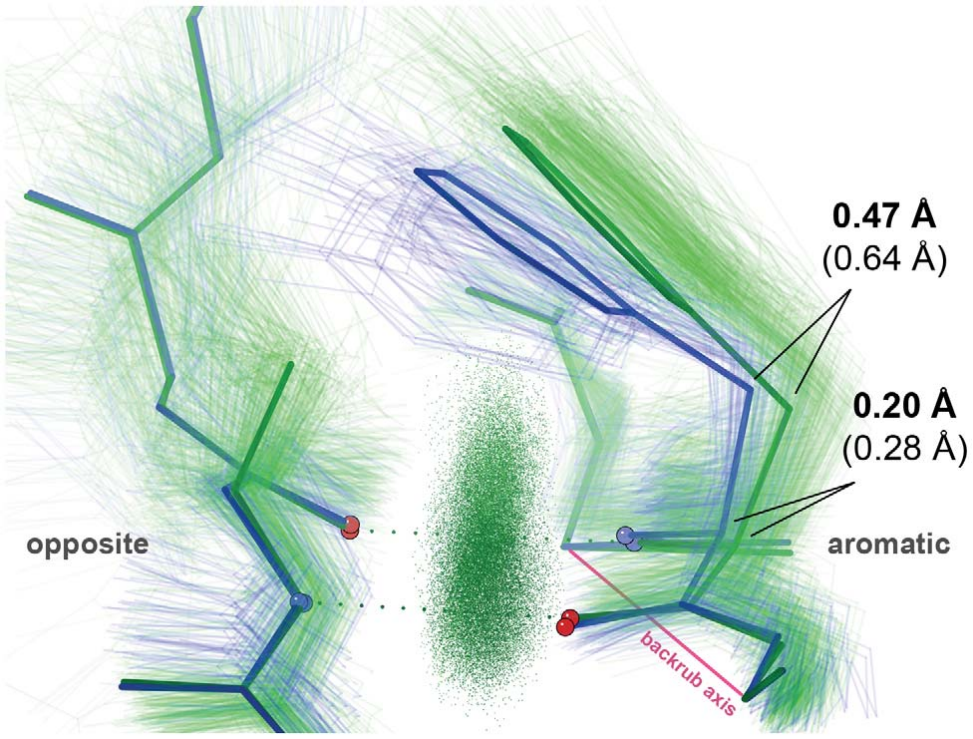
Backrubs at aromatic residues in antiparallel β-sheet. Crystal structure ensembles for Phe/Tyr across from Gly (light blue) vs. anything else (light green) are related by a backrub. Lowest-energy BRDEE conformations for 1z84 Phe171 across from Gln188 (visually truncated at C_β_ for clarity) (dark green) vs. Gln188→Gly (dark blue) have a similar relationship. Aromatic C_α_ and C_β_ displacements for both average crystal structures (lighter, in parentheses) and low-energy BRDEE conformations (darker) evoke a hinge-like backrub operation. Ensemble mainchain-mainchain H-bonds are illustrated with “pillows” of green all-atom contact dots [44].

In 72 examples the amino acid on the opposite strand is a Gly, in which case the aromatic sidechain moves downward to contact the Gly C_α_ H. In the other 249 examples the C_β_ H atoms of the amino acid on the opposite strand push the aromatic ring upward (Figure 4, Table 2, Dataset S2 kinemage). Pro cannot provide both β H-bonds, but all other non-Gly residues are equivalent in this role, since their sidechains must avoid the aromatic ring and present only C_β_ H atoms toward it. The average C_β_ deviation from ideality (0.05–0.06 Å) is far less than the outlier threshold (0.25 Å) [23], and the average change in aromatic C_α_-C_β_-C_γ_ bond angle is very small (0.6°, <1σ) (Figure S3 in Text S1), resulting in a <0.05 Å shift of the C_ζ_ contact point. These observations argue against the possibility that this large movement of the planar aromatic group is produced just by a bond-angle “hinge” with C_α_ or C_β_ as the pivot. Rather, a dipeptide backrub rotation of about 11° (presumably 5–6° from neutral in each direction) almost perfectly interrelates the two average conformations.

**Table 2:** Backrub changes at β aromatics across from Gly vs. other.

Structures	Average Crystal	BRDEE 1gyh A	BRDEE 1khb A	BRDEE 1z84 A
Aromatic	F	Y109	F144	F171
Opposite	G→A	G122→[A]	G157→[A]	Q188→[G]
ΔC_α_ *i*−1 (Å)	0.01	0.02	0.01	0.01
ΔC_α_ *i* (Å)	0.28	0.25	0.24	0.20
ΔC_α_ *i*+1 (Å)	0.09	0.03	0.01	0.02
ΔC_β_ *i* (Å)	0.64	0.50	0.51	0.47
ΔC_ζ_ *i* (Å)	1.34	0.96	1.05	1.01
Backrub (°)	−11	−10	−11	−11
Δτ *i*−1 (°)	−0.2	−0.5	−0.4	+0.3
Δτ *i*+1 (°)	+1.0	+2.8	+1.5	+1.2

Distances are after superposition into the same reference frame using 5 C_α_s (aromatic *i*−2, *i−1*, *i*+1, *i*+2 and opposite *i*).

Distances for BRDEE for atoms at or beyond Cα *i*±1 are shown, as opposed to the N-cap case, because superposition into the same reference frame is subtly affected by allowing backrubs at the opposite C_α_ in BRDEE.

The signs of the backrub rotation angles and Δτ values are in terms of across-from-Gly→across-from-other.

For average crystal structures, average sidechains (based on average C_β_ positions and χ dihedral angles) were added in KiNG. The τ value used for each Δτ is an average across the crystal structure ensemble, to be consistent with the methodology for N-caps.

For input to BRDEE, each example was used twice: first with its original deposited sidechain on the opposite strand, and then with a fully ideal sidechain of the opposite type (Gly if originally not Gly, Ala if originally Gly) added in KiNG (residue names in [brackets]). The τ value used for each Δτ is taken directly from the lowest-energy computed structure.

#### β aromatic BRDEE computation

Fewer examples, and more variation for β-sheet than for α-helix conformation, prevented the ideal-start calculation used for the N-cap case. Instead, low-energy conformations were computed by BRDEE for several examples judged to be appropriately representative of their respective type (across from Gly or across from other) (see Methods). In all cases, the lowest-energy conformation appears to match the average crystal structure very well, whether across from Gly or from some other amino acid with a C_β_ atom (Figure 4). Computed shifts in C_α_, C_β_, C_ζ_ position between the computed wildtype and mutant structures are similar to shifts between the two crystal structure populations, albeit systematically a bit smaller (Table 2, Text S1). As with N-caps, though to a slightly lesser extent here in extended β conformation, *in silico* backrubs place more strain into the *i*±1 τ angles than do real structures (Table 2); however, the discrepancies are on the same order as the standard deviations of the experimental τ distributions, which are around 3° (data not shown).

These results confirm both that BRDEE in conjunction with a simple force field reproduces natural conformations well and also that backrubs model the relationship well.

## Discussion

### Backrubs and Natural Protein Evolution

It is known that backrubs relate conformations that interchange dynamically [9]. In this study we further show that, at least for certain specific motifs, backrubs relate conformations that “interchange” based on point mutations. For the β aromatic motif, local restraints on steric packing influence the aromatic residue's backbone indirectly via the other altered sidechain. This is in contrast to helix N-caps where the sequence change and the backrub occur at the same residue, as seen previously for rotamer changes [9]. Taken together, these findings support the intriguing idea that backrubs may “foster” mutations, easing them into the structure and promoting their survival into future generations. In this paradigm, backrubs enable individual mutations that provide the raw material for natural selection.

The two specific motifs analyzed here represent only about 0.5% of the protein residues in our Top5200 data set, and thus in one sense the scope of this study is relatively narrow. However, a tight focus was necessary to substantiate the idea of mutation-coupled backrubs with sufficient certainty, due to the coordinate error problem in the alternative approach of comparing individual wildtype and mutant crystal structures directly. These two cases were chosen as common, well-defined motifs where the primary interaction environment of the changing sidechain is provided by local secondary structure and is therefore consistent across hundreds of examples. For the general case of an individual mutation, the potential interaction environment is also the same before and after; however, it is seldom simple enough to be closely repeated in numerous proteins. Furthermore, as shown in previous work at ultra-high resolution [9], 2/3 of alternate conformations that move C_β_ demonstrate backrubs between rotamers of the same amino acid. All in all, therefore, it is reasonable to assume that the general prevalence of backrub accommodation at sites of mutation is significantly higher than the “lower bound” provided in this study.

The backbone shift considered on its own is continuous and low-energy, without a barrier, while the two-state behavior is contributed by the sidechain switch between rotamers, between H-bond partners, or between amino acids. In the dynamics case of jumps between distinct sidechain rotamers or H-bonds [9], both conformations are quite favorable, but backbone and sidechain must change together. In the evolutionary case, such as the single amino acid changes between stable motifs illustrated in this paper, the two amino acid types cannot coexist in the same molecule. The sidechain-backbone coupling shifts the energy landscape for the backbone [31], stabilizing a different choice within a shallow energy well. From a modeling perspective, examination of existing fast-timescale structural dynamism may illuminate other possibilities for mutations on an evolutionary timescale [32].

Mutation-coupled backrubs are small local changes, which presumably mediate neutral drift much more often than they aid large-scale structural rearrangements or changes in function. However, the accumulation of changes via neutral drift over time may in fact enable future large-scale changes by subtly altering the native state energy landscape such that eventually a tipping point is reached. Recent analysis of the evolution of an ancient protein confirms that some function-altering mutations required structural pre-stabilization by earlier “permissive” mutations [33]; backrubs may facilitate such preemptive sequence changes by shifting the backbone such that the functionally neutral amino acids can fit. Backrub-related sequence changes could also sometimes enable functional change based on a purely local adaptation when they occur in active sites, either directly or by first enhancing functional promiscuity.

Note that we do not directly address true evolutionary relationships between proteins in this study. Rather, we substantiate the idea that backrubs enable single amino acid changes at specific motifs, which could aid actual evolution within a protein family [15].

### Backrubs and Computational Protein Design

It is only natural to segue from the role of backrubs in protein evolution to their utility for protein design – essentially a computational analog of molecular evolution. Our results indicate that, despite the relative simplicity of their functional form, molecular-mechanics-based force fields like Amber plus EEF1 that are commonly used for protein design can in fact accurately recapitulate empirically-observed backbone conformation for multiple specific structural motifs, given the chance to access them via a backrub. (Note that the cases presented here were dominated by single interactions such as H-bonds or steric packing; a higher-cost energy function might be needed to maintain similar accuracy if different interactions are competing and need to be compared quantitatively.)

Thus, predicting the conformational consequences of a sequence change in computational protein design is in large part a search problem: if the appropriate regions of protein conformational space are searched efficiently, in many cases low-cost energy functions can do the rest. Unfortunately, that space is vast indeed even for a single sequence, as we know from Levinthal's famous thought experiment [34]. The additional consideration of combinatorial mutations (even when conformational changes are restricted to simple sidechain rotamer alternatives) creates a space that is even more difficult to search, as shown by the proof that protein design is *NP*-hard [35]. Of course, backbone flexibility further enlarges the search space.

However, flexible-backbone design algorithms like BRDEE are excellent candidates for this task in many cases because (1) they are based on empirically demonstrated types of flexibility and (2) they come with mathematical guarantees of their accuracy with respect to the input parameters. Other algorithms that search over amino acid and rotamer identities, then minimize over backrub degrees of freedom *post facto* are not guaranteed to identify the global minimum energy conformation (GMEC) given the input model (starting structure, rotamer library, energy function). An advantage of BRDEE is that it incorporates backrub minimization awareness directly into the amino acid and rotamer comparison stages of dead-end elimination, and thus is guaranteed to identify the GMEC given the input model. Because BRDEE avoids becoming trapped in local minima, it effectively decouples the often intertwined issues of conformational search and scoring. Therefore, as a result of using BRDEE, this paper gives the limit of how well any algorithm can perform given our input model. In the future we plan to implement additional empirically-validated small backbone motions, such as peptide flips and tripeptide shears [9], [14], to improve coverage of conformational space.

### Conclusion

Overall, we have demonstrated that the backrub, a model of local backbone motion previously only documented for dynamic rotamer changes, also applies to local sequence changes. This finding is an important direct validation for the application of the backrub to the study of natural protein evolution and to continuing efforts in computational protein design.

## Methods

### Creation of Structure Database

To identify numerous examples of the desired motifs, we used a “Top5200” database of high-quality protein structures. The rapid growth of the Protein Data Bank (PDB) [36] in recent years enabled the creation of a high-quality database with an order of magnitude increase in size relative to the previously described Top500 [23] while maintaining similar standards of resolution and structure quality. However, due to sheer logistics it also necessitated a more automated selection protocol.

We included at most one protein chain per PDB 70% sequence-similarity cluster as of April 5, 2007. We chose the representative for each such cluster as the chain with the best average of resolution and MolProbity score [37] where resolution is <2 Å. MolProbity score is an estimate of the resolution at which a structure's steric clashes, rotamer quality, and Ramachandran quality would be average; thus the average of resolution and MolProbity score is a combined experimental and statistical indicator of structural quality [37]. The homology filter prevents redundancy and thus over-representation of certain motifs or substructures.

To calculate the MolProbity score for each chain, first hydrogens were added with the program Reduce [38]. The -flip flag was used in order to allow Asn/Gln/His flips throughout the structure, including at interfaces where multimer partners may participate in hydrogen-bonding networks. All protein chains with at least 37 residues were then extracted, along with any “het” atoms or waters with the same chain identifier, and MolProbity score was calculated for each chain.

Two “post-processing” steps were required. First, we removed four chains whose PDB structures had been obsoleted and replaced them with updated structures where possible (1sheA→2pk8A, 1wt4A→2v1tA, 2eubA→2pl1A, 2f4dA→no replacement). Finally, we removed two chains with incomplete or unclear PDB files (1c53A had only C_α_ atoms, 3ctsA had only “UNK” unknown residue types). The resulting 5199 protein chains make up about a million residues.

### Identification of Motifs

#### Identification of α-helix N-caps

We first noticed that backrubs may explain backbone adjustments upon mutation between short and long N-caps sidechains while examining N-caps in T4 lysozyme. Visual analysis using the BACKRUB tool in KiNG [39] revealed that a modest backrub (about 7°) nicely models the relationship between the Thr59 N-cap in the wildtype structure (2lzm) and the Thr59→Asn N-cap in the mutant structure (1lyg) [40]. Intriguingly, as well as being one of the most common N-caps, Ser is the most common amino-acid type for backrubs between alternate conformations in crystal structures [9], perhaps because it has many distinct possibilities for sidechain-backbone H-bonding.

N-caps were identified using a helix recognition algorithm based loosely on DSSP [41] that combined consecutive α-like (*i*+4 mainchain-mainchain H-bond) and/or 3_10_-like (*i*+3 mainchain-mainchain H-bond) helical turns. To approximate the visually determined definition of an N-cap as the first residue within the C_α_ cylinder of the helix [17] but extend it to a much larger data set, residues were added to the N-terminus as putative N-caps if the distance from their C_α_ to the *i*+3 C_α_ was less than 5.9 Å. We chose this distance cutoff in order to include true N-caps at helix ends that were somewhat but not excessively stretched, i.e. with relatively weak mainchain-mainchain H-bonds but within the helix C_α_ cylinder and close enough to contribute with a sidechain-mainchain capping interaction. No amendments to the DSSP algorithm were made for C-caps, which we did not specifically examine in this study. Helices were required to include at least five residues including N- and C-caps.

We also took precautions to help offset any leniency in helix extension introduced in the steps described above. To ensure that a tightly defined subset of N-caps was being examined, H-bond pseudo-energies were computed as in DSSP with the standard −0.5 kcal/mol cutoff. N-caps with an *i*+4 but not an *i*+3 mainchain-mainchain H-bond were labeled α, N-caps with an *i*+3 but not an *i*+4 mainchain-mainchain H-bond were labeled 3_10_, and N-caps with both *i*+3 and *i*+4 mainchain-mainchain H-bonds were labeled bifurcated α/3_10_ and were ignored in this study.

For N-cap sequence propensity analysis, all α and 3_10_ N-caps of each amino acid type were compared to the general case of all residues in the Top5200 regardless of backbone conformation. For N-cap backrub analysis, on the other hand, only α N-caps with *i*+3 but not *i*+2 sidechain-backbone H-bonds were used. These N-caps are found in the “extended” region of Ramachandran space, with two peaks of occurrence near (−150°,170°) and (−80°,170°) [42]. We required φ,ψ values near the “right-most” of these peaks, i.e. between (−100°,155°) and (−60°,180°), because it is the more populated of the two peaks for both the Asn/Asp and Ser/Thr groups and because the restriction provides a consistent depiction of the subset of N-caps that could undergo backrubs to relate to each other.

As described in Results, we also created two control categories. For the “other N-caps” category, the criteria were the same as for Asn/Asp or Ser/Thr N-caps, except we required some other amino acid identity at the N-cap position. For the mid-helix category, we required strictly α-helical φ,ψ values between (−65°,−45°) and (−55°,−35°) for the central residue of interest, at least four residues labeled “H” for α-helix by DSSP [41] in each direction, and at least 13 such residues total in the helix. For the Ser/Thr, Asn/Asp, and other N-cap categories, the N-cap residue was required to have maximum mainchain or sidechain atom B-factor <40 [23]. For the mid-helix category, the maximum B-factor was 20. To attain roughly similar sample sizes for all four categories, we randomly selected 500 examples for Ser/Thr (out of 3,208) and for mid-helix (out of 17,047), to match the 429 Asn/Asp and 538 “other” N-caps.

Examples of the N-cap motif were superposed onto one another using the N-cap *i*−1 and *i*+1 to *i*+3 C_α_s; the N-cap *i* C_α_ itself was not used for superposition to avoid biasing its final average position. For the mid-helix control category, only the *i*+1 to *i*+3 C_α_s were used, because there is no structural equivalent to the N-cap *i*−1 residue. Root mean square deviation (RMSD)≤1 Å relative to an ideal reference N-cap (see BRDEE input below) for Asn/Asp, Ser/Thr, and other N-caps, or relative to an ideal poly-Ala helix (similar to BRDEE input) for mid-helix, was required to keep each example.

Average motifs were then generated by taking the mean position of all backbone atoms (including hydrogens) and C_β_ atoms from N-cap *i*−1 to *i*+3. A Ser rotamer with χ_1_ = +65° and an Asn rotamer with χ_1_ = +66° and χ_2_ = +30° were added to reflect median dihedral angles from the data set, while maintaining the average C_β_ positions. Finally the average structures were superposed onto one another, again using the N-cap *i*−1 and *i*+1 to *i*+3 C_α_s (*i*+1 to *i*+3 C_α_s for mid-helix).

Using the same protocol, we also analyzed 388 Asn/Asp and 976 Ser/Thr examples from the second Ramachandran peak, near (−150°,170°) instead of (−80°,170°) as mentioned above. The *i*−1 C_α_ in the secondary cluster is displaced by about 1 Å relative to the primary cluster due to the differing φ values (−150° vs. −80°) before entering the helix; thus, given the magnitude of the backrub motion we hoped to capture, it was unreasonable to compute an average structure for the combined set. However, separate analysis of the secondary cluster revealed a very similar backrub relationship as seen in the primary cluster (Dataset S1 kinemage, PDB files in Dataset S3).

#### Identification of aromatics in antiparallel β-sheet

For β aromatics, we identified Phe/Tyr residues with “plus” χ_1_ (0–120°) in antiparallel β-sheet. The “opposite” residue is in the direction of the aromatic sidechain, between the narrow pair of mainchain H-bonds. Both residues were required to have maximum mainchain or sidechain atom B-factor less than 40, and to have at least one additional β residue (according to the custom DSSP-based secondary structure identification algorithm described above) in each direction along their respective strands.

To avoid irregularities from strand ends, we defined the “fray” parameter f:


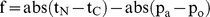


where t_N_ (N-ward twist) is the dihedral between C_α_ *i*−1 and *i* on the aromatic strand and C_α_ *i* and *i*+1 on the opposite strand; t_C_ (C-ward twist) is the dihedral between C_α_ *i* and *i*+1 on the aromatic strand and C_α_ *i* and *i*−1 on the opposite strand; p_a_ (aromatic pleat) is the angle between C_α_ *i*−1, *i*, and *i*+1 at the aromatic residue; and p_o_ (opposite pleat) is the angle between C_α_ *i*−1, *i*, and *i*+1 at the opposite residue. A difference in twist indicates that the two strands locally “pull apart” from each other; any difference in amount of pleating between the two strands is subtracted as a correction term. Fray was constrained to less than 10° for our motif search.

Examples of the β aromatic motif were superposed onto one another using the aromatic *i*−2, *i*−1, *i*+1, *i*+2 C_α_s and the C_α_ opposite *i* as an “anchor” point; the aromatic *i* C_α_ itself was not used for superposition to avoid biasing its final average position. RMSD≤1 Å relative to a reference example was required to keep each example. For each category (across from Gly vs. other), 10 arbitrarily chosen reference examples were tried; the one that resulted in the fewest examples being pruned by the RMSD≤1 Å filter was deemed representative of its category (1gyhA Tyr109/Gly122 pruned only 3.8% of across-from-Gly examples, and 1avbA Phe6/Phe221 pruned only 4.2% of across-from-other examples). The observed backrub relationship was similar when manually selected reference examples judged to representative of each category or completely randomly selected reference examples were used instead (data not shown), and the RMSD between the representative reference examples described above (0.11 Å) was significantly less than the 1.0 Å RMSD filter, so it is unlikely the choice of particular reference examples pre-determined the result of an observed backrub between the sets of examples superposed onto the reference examples.

Average motifs were then generated by taking the mean position of all backbone atoms (including hydrogens) and C_β_ atoms from residues *i*±2 on both strands. Average Phe rotamers with χ_1_ = +65° and χ_2_ = +80° for the across-from-Gly category and with χ_1_ = +66° and χ_2_ = +84° for the across-from-other category were added to reflect median dihedral angles from the data set, while maintaining the average C_β_ positions. Finally the average structures were superposed onto one another, again using the aromatic *i*−2, *i*−1, *i*+1, *i*+2 C_α_s and the C_α_ opposite *i*.

### Computational Design

For all BRDEE calculations, we used the same input parameters as described previously [10] for energy function, rotamer library, τ filter, etc. Briefly, the energy function consists of the Amber electrostatic and van der Waals terms [24] plus the EEF1 pairwise solvation energy term [25]. The τ filter prevents large strain at the only bond angle allowed to change in the calculations [10].

#### Computational design of α-helix N-caps

For N-cap calculations, we started from an ideal helix (φ,ψ −60°,−40°) with poly-Pro conformation (φ,ψ −80°,170°) for the N-cap and its preceding residue. All residues were Ala except for the N-cap, which was either Asn or Ser. At the N-cap position, primary backrub rotation angles from −15° to +15° in increments of 0.5° were allowed, and the default ε factor of 0.7 was used for back-rotation of the single peptides. At the N3 position, backrubs were not allowed as part of the main search. However, using KiNG [39] we manually determined that single-peptide rotation angles from −5° to +15° of the N3 peptide could help optimize the H-bonding angle of its backbone amide to the N-cap sidechain H-bonding angle and were viable in terms of sterics, Ramachandran dihedrals [23], and τ angles; therefore we included such a rotation in increments of 1°.

#### Computational design of aromatics in antiparallel β-sheet

For β aromatic calculations, the strand twist and curl vary too much to construct averaged cases. Instead, we repeated the computational experiment several times with individual examples of the motif from different experimental structures as input coordinates. Representative examples such as 1khbA Phe144-Gly157 and 1z84A Phe171-Gln188 were chosen because they were visually relatively “middle of the pack” and were not obvious outliers in terms of geometric parameters like fray. Primary backrub rotation angles from −15° to +15° in increments of 0.5° were allowed at both residue positions, with the default ε factor of 0.7. (Backrubs at the opposite position were of less interest for this study, and indeed turned out to be much smaller than at the aromatic position.) If the original opposite residue was Gly, BRDEE was run for both the original coordinates and a Gly→Ala mutant. Likewise, if the original opposite residue was anything but Gly, BRDEE was run for both the original coordinates and an Xaa→Gly mutant.

### Visualization

The KiNG graphics program [39] was used both to study the superposition results and to produce the figures. Dataset S3 provides raw PDB coordinate files for N-cap and aromatic crystal structure examples, average crystal structures, and BRDEE lowest-energy models.

## Supporting Information

Dataset S1 This is a plain text kinemage graphics file allowing interactive, three-dimensional exploration of the N-cap conformations from Figure 3. It can be viewed locally with the free program KiNG (http://kinemage.biochem.duke.edu/software/king.php) either by renaming the file to have a “.kin” file extension or by keeping the current filename but using the “All files” format option. Alternatively, it can be viewed on the web by uploading to MolProbity (http://molprobity.biochem.duke.edu/) as a kinemage file and selecting “View in KiNG”.
(TXT)

Dataset S2 This is a plain text kinemage graphics file allowing interactive, three-dimensional exploration of the aromatic conformations from Figure 4. It can be viewed locally with the free program KiNG (http://kinemage.biochem.duke.edu/software/king.php) either by renaming the file to have a “.kin” file extension or by keeping the current filename but using the “All files” format option. Alternatively, it can be viewed on the web by uploading to MolProbity (http://molprobity.biochem.duke.edu/) as a kinemage file and selecting “View in KiNG”.
(TXT)

Dataset S3 This is a compressed tar archive containing lists of all individual examples and PDB coordinates for average and calculated structures (a README file explains the naming conventions) for both the crystal-structure ensembles and the BRDEE calculated structures.
(GZ) Click here for additional data file.

Text S1 This file provides a discussion of the differences between α-helix N-caps and 3_10_-helix N-caps, and compares and contrasts in more detail the average crystal structures and low-energy BRDEE models for both the N-cap and β aromatic cases.
(DOC)
