# Supplementary material for: The Role of Local Backrub Motions in Evolved and Designed Mutations
Source: PLoS Comput Biol. 2012 Aug 2;8(8):e1002629. doi: 10.1371/journal.pcbi.1002629 (PMC3410847; doi:10.1371/journal.pcbi.1002629)
Supplement: Text S1 — This file provides a discussion of the differences between α-helix N-caps and 310-helix N-caps, and compares and contrasts in more detail the average crystal structures and low-energy BRDEE models for both the N-cap and β aromatic cases. (DOC) [file pcbi.1002629.s004.doc]

Comparison of α-Helix N-caps to 3_10_-Helix N-caps

3_10_-helix N-caps are relatively permissive in terms of amino acid identity, except for the approximately 2.5-fold spike of preference for Pro (Figure 2). Such motifs may be favorable due to van der Waals interactions between the Pro ring and the *i*+2 and *i*+3 sidechains of the first turn, as seen in Figure S1. The majority of 3_10_ Pro N-caps have β φ,ψ (86%, 403/471), which presents the Pro sidechain toward the first turn. Nearly all (96%) of those proline rings make van der Waals contact with first-turn sidechains.


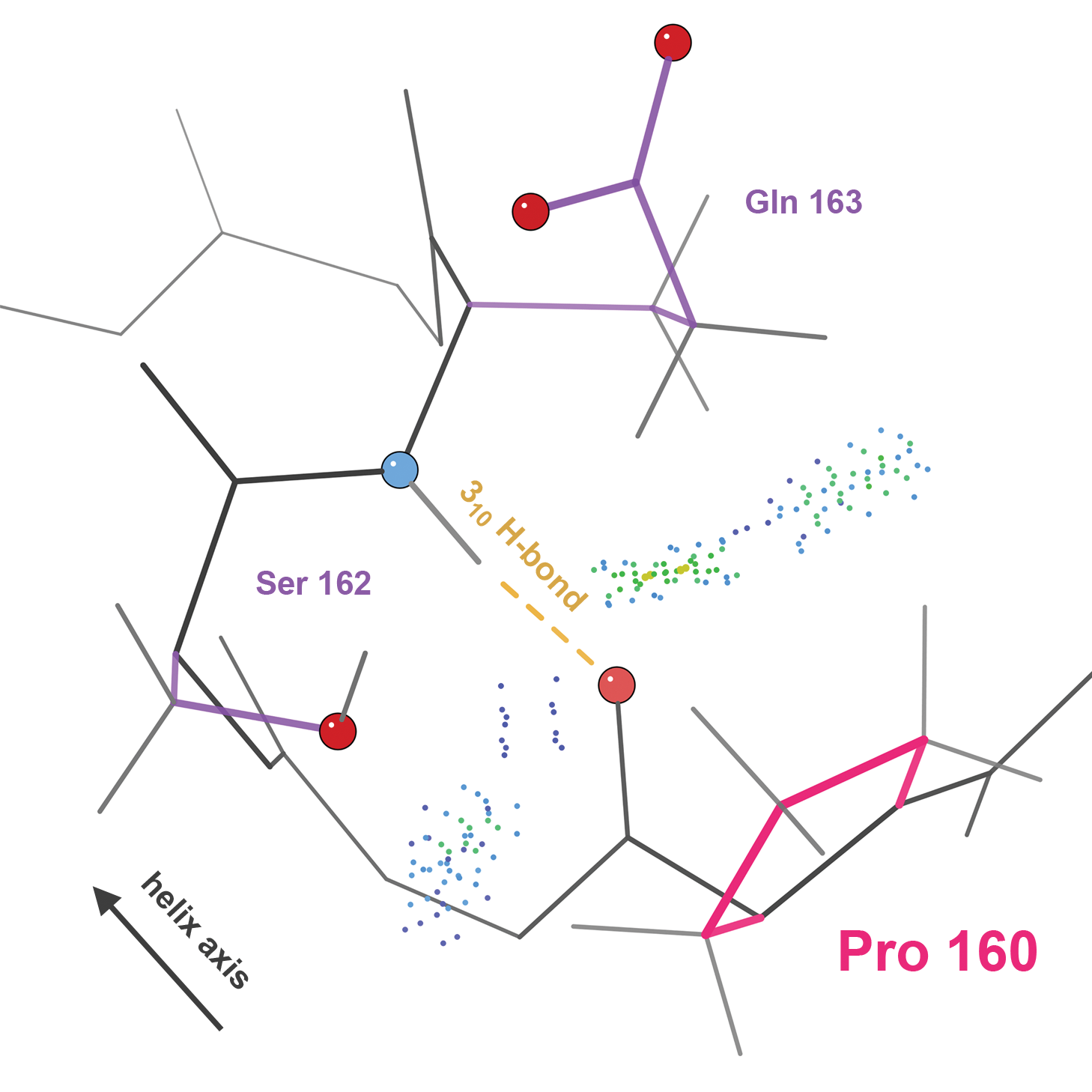


**Figure S1: An example of the 3_10_ Pro N-cap motif.**  The ring of Pro 160 (pink) in 1m79 chain A makes van der Waals interactions (green/blue dots) with the sidechains of Ser 162 and Glu 163 (purple). These close interactions are possible because the 3_10_-helical *i*+3 mainchain-mainchain H-bond (gold dashed line, pale green dots) pulls the Pro close enough to the first-turn sidechains. Note that Ser at position *i*+2 and Glu at position *i*+3 are among the most common identities given a 3_10_ Pro N-cap.

Perhaps not coincidentally, Pro is also favored at the *i*+1 position immediately following an α-helix N-cap . Proline is good in the first turn of any helix type, because the ring interferes sterically with any potential preceding helix turn, removes one unsatisfied NH, and entropically favors helical φ,ψ because it has fewer other possibilities than other residues. Pro slightly prefers a preceding residue that is not in helical conformation, so it is more common in N-cap *i*+1 than *i*+2 or *i*+3, but it cannot make either the N-cap sidechain-mainchain H-bond or the reciprocal "cap-box" *i*+3 sidechain H-bond to the N-cap NH , so it is rare as an a-helix N-cap. That is presumably not a disadvantage for 3_10_-helix where those interactions do not occur, so Pro is well suited as a 3_10_ N-cap.

This suggests the possibility that helix N-termini can transform from more tightly-wound 3_10_ (3 residues/turn) to looser α-helix (3.6 residues/turn), or vice versa, by deletions or insertions in the preceding loop. For example, a canonical α N-cap motif, with Asn/Asp/Ser/Thr at position *i* and Pro at position *i*+1, could be transformed to a 3_10_ Pro N-cap motif if deletion of residues in the preceding connection/loop pulled the first turn tighter. There is no evidence of an increased prevalence (relative to general-case protein structure) of vestigial Asn/Asp/Ser/Thr at the *i*-1 position immediately preceding extant 3_10_ Pro N-caps (data not shown), suggesting that such residues may change identity once no longer useful. Conversely, a 3_10_ Pro N-cap could be the precursor to a canonical α N-cap motif if insertion of residues in the preceding connection/loop added “slack” to the first turn, in which case the Pro would become the N-cap *i*+1 and the preceding residue would become the new α N-cap. Either before or after the insertion and conformational change, this residue could mutate to Asn/Asp/Ser/Thr to solidify the new α N-cap motif.

Also note that Asn/Asp are preferred at 3_10_ N-caps but Ser/Thr are neutral, despite both being strongly preferred at α N-caps (Figure 2). As mentioned in the main text, with α N-cap conformation both categories of sidechains form strong *i*+3 sidechain-backbone H-bonds, but with 3_10_ N-cap conformation this interaction is not possible. Asn/Asp instead form strong *i*+2 sidechain-backbone H-bonds, and are therefore still preferred as 3_10_ N-caps (albeit slightly less so than as α N-caps). Ser/Thr, on the other hand, are slightly too short and form only weak *i*+2 sidechain-backbone H-bonds, and are therefore not preferred as 3_10_ N-caps. However, they are not disfavored either; these net neutral 3_10_ N-cap preferences suggest that any unfavorable energetics may be counteracted by the small benefit enacted by the weaker *i*+2 H-bonds.

Backrubs of α-Helix N-caps

The orientation of the peptide between residues *i*+2 and *i*+3, containing the H-bonding amide group, does not deviate significantly in the N-cap crystal structures relative to its “equilibrium” mid-helix crystal structure orientation. However, this peptide was allowed to rotate slightly in the BRDEE calculations in order to optimize the N-cap H-bond interaction; as a result, it systematically tilts inward toward the central helical axis by 7-9˚ in the lowest-energy computed models as compared to the crystal structures. This may indicate a limitation of the primarily electrostatic Amber hydrogen bonding potential currently employed in BRDEE ; future work could involve replacing Amber with CHARMM or another molecular mechanics force field to probe this subtlety. Regardless, the small discrepancy at the *i*+2 to *i*+3 peptide does not detract from the clear observations of backrub-like backbone shifts at the N-cap position.

The Ser/Thr → Asn/Asp C_α_ displacement is similar for average crystal structures and computed structures, but the C_β_ displacement is slightly greater for the former than the latter (Table 1, Dataset S1 kinemage). This deviation from full-sidechain hinge-like displacements reveals that the average crystal structure Ser/Thr C_β_ flexes slightly forward and the average crystal structure Asn/Asp C_β_ flexes slightly backward, relative to an ideal C_β_ position , to maintain a similar *i*+3 sidechain-mainchain H-bond length. However, only two Asn/Asp examples and no Ser/Thr examples examined have C _β_ deviations above the 0.25 Å outlier limit (relative to an ideal C _β_ position) . For perspective, the average C_β_ deviation observed for both categories (0.08 Å) shifts the N-cap sidechain H-bonding oxygen by < 0.15 Å for both categories (0.13 Å for Asn/Asp, 0.12 Å for Ser/Thr), but 11˚ backrubs (see Table 1) shift the same oxygen by over 0.60 Å for both categories (0.64 Å for Asn/Asp, 0.69 Å for Ser/Thr). The bond angle flexibility centered at the C_β_ in the C_α_-C_β_-C_γ_ angle is also very small: < 1.0˚ for Asn/Asp N-caps relative to general-case Asn/Asp and < 0.4˚ for Ser/Thr N-caps relative to general-case Ser/Thr (< 1σ for both categories) (Figure S2). Thus bond angle flexibility may play some role in accommodating sequence changes at this motif, but the backrub has a markedly greater effect.

Note that the discrepancies between Δτ for computed vs. experimental structures are not excessive compared to the standard deviations of the experimental τ distributions, which are around 3˚ (data not shown). Also, the Δτ changes are greater at N-caps, particularly at the more helical *i*+1 position (Table 1), than at β aromatics (Table 2); this differential bond angle strain may help explain the observation that backrubs are more common in extended structure than in helical structure [5].


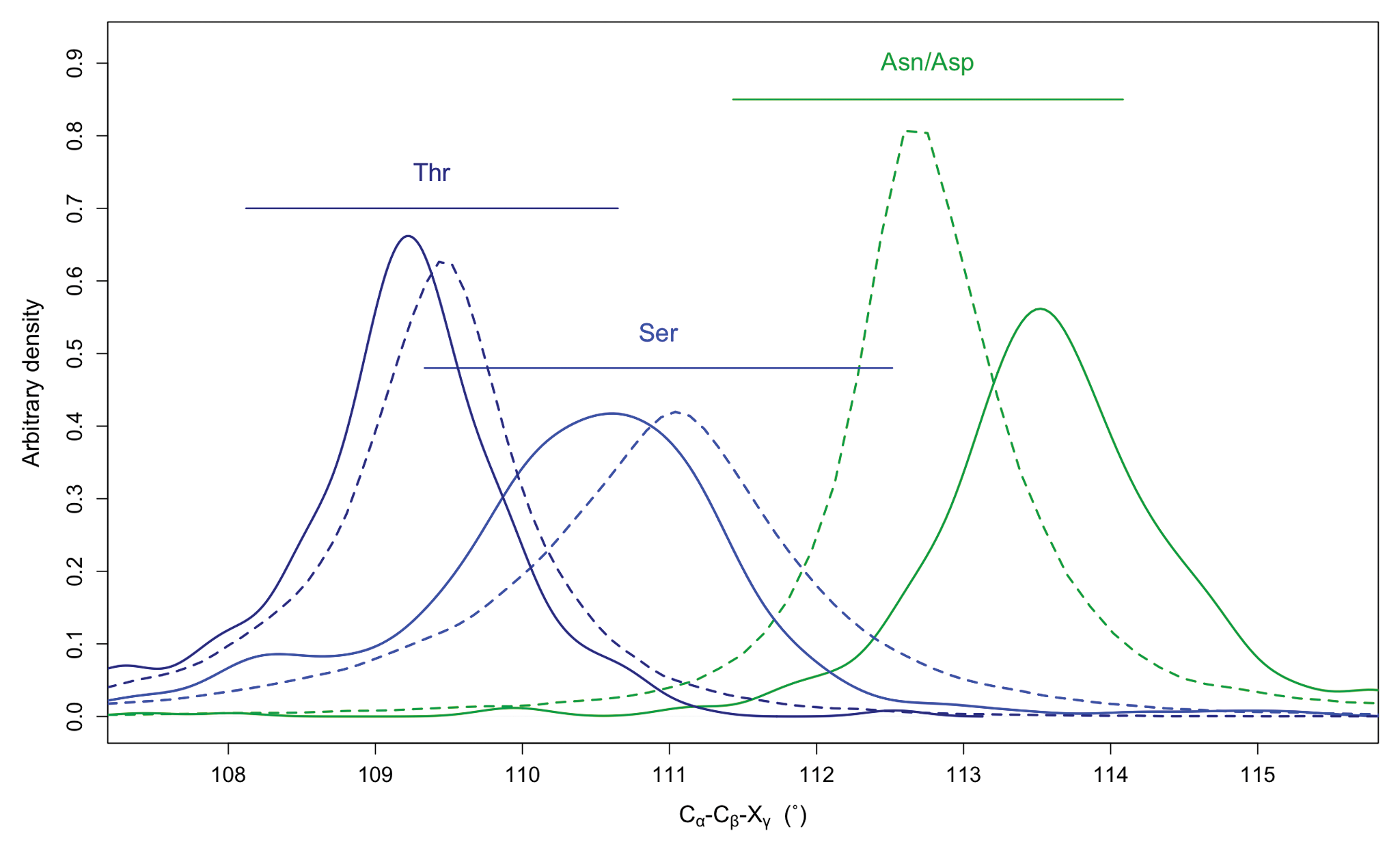


**Figure S2: Sidechain C_α_-C_β_-C_γ_ or C_α_-C_β_-O_γ_ bond angles are not unusual at N-caps.** The median for the distribution for Asn/Asp N-caps (same data set as in Figure 3) (green solid curve) is within ± 1 standard deviation (green horizontal line segment) of the median for the general distribution for Asn/Asp in the Top5200 (green dotted curve). Similarly, the median for the distribution for Ser N-caps (Ser subset of same data set as in Figure 3) (lighter blue solid curve) is within ± 1 standard deviation (lighter blue horizontal line segment) of the median for the general distribution for Ser in the Top5200 (lighter blue dotted curve), and the median for the distribution for Thr N-caps (Thr subset of same data set as in Figure 3) (darker blue solid curve) is within ± 1 standard deviation (darker blue horizontal line segment) of the median for the general distribution for Thr in the Top5200 (darker blue dotted curve). The curves shown here and in Figure S3 are the result of kernel density estimation using Gaussian smoothing kernels with bandwidths automatically determined based on Silverman’s rule of thumb [6], as implemented in the default “density” function in R [7].

Backrubs of Aromatics in Antiparallel β-Sheet

For β aromatics as well as for N-cap backrubs, bond-angle changes at C_α_ and C_β_ are small (see Figure S3). As mentioned in the main text (Table 2), the positional shifts in aromatic C_α_, C_β_, C_γ_ atoms when opposite Gly vs. anything else are in the same direction in the lowest-energy computed models and crystal structures, but they are smaller in the former than the latter. This may be due in part to the use of an older version of Amber [8] with imperfect parameters, but a more likely explanation is the inclusion of backrubs at the opposite residue as well as the aromatic residue (see Methods). In 2/3 examples where the opposite residue was Gly (either wildtype or mutated to Gly), the computed backrub moved it “inward” toward the aromatic residue, thus helping maximize favorable contact (in the third case the backrub was 0°). In 2/3 examples where the opposite residue was something other than Gly (either wildtype or mutated from Gly to Ala), the computed backrub moved it “outward” away from the aromatic residue, thus helping minimize unfavorable contact. In the third example, the opposite residue is 1z84 Gln188; χ_1_ stays nearly the same, but for the lowest-energy computed model (used in the main analysis), χ_2_ changes to pack the Gln amide group underneath the aromatic ring and allow an “inward” backrub (Dataset S2 kinemage). The second-lowest-energy computed model, on the other hand, packs the amide against the aromatic ring edge like the crystal structure, and has an “outward” backrub.

It seems clear that the gain in favorable inter-strand contact is more efficient for backrubs at the aromatic side, because there is more leverage out to the ring than just for a Gly or C_β_. In our computational experiments, there is no explicit penalty for backrubs away from relaxed β structure, so long as the τ bond angle changes are not excessive (see Methods). Given that the natural proteins undergo small or no opposite-strand backrubs, perhaps having a modest penalty would be more realistic. Even so, in all 3 comparison examples the computed backrub at the aromatic residue was indeed larger than at the opposite residue, so our conclusion that aromatic residues undergo backrubs to accommodate cross-strand mutations is substantiated as the major effect.


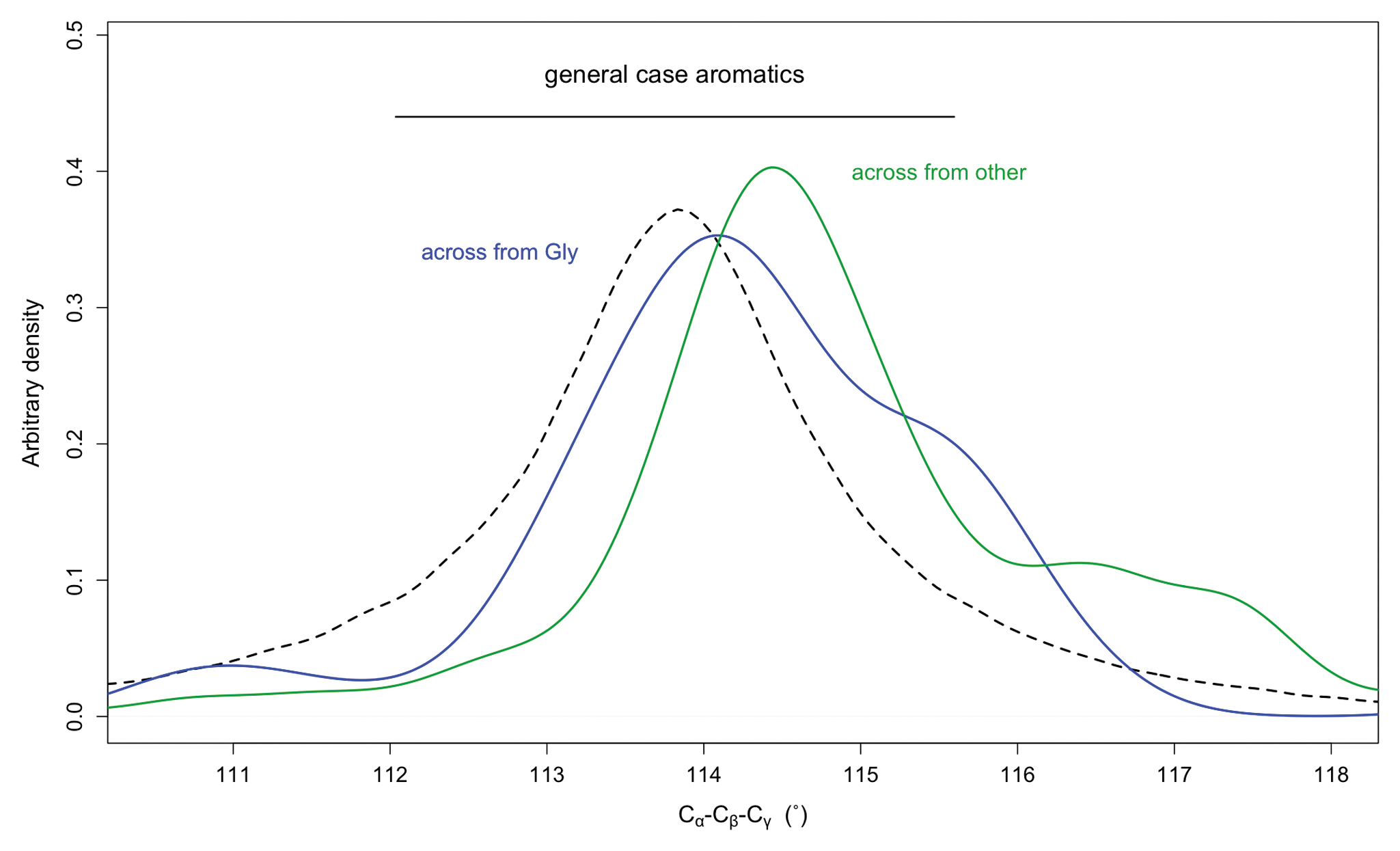


**Figure S3: Sidechain C_α_-C_β_-C_γ_ bond angles are not unusual at antiparallel β-sheet aromatics with “plus” χ_1_ rotamers.** The distributions for aromatics across from Gly (same data set as in Figure 4) (blue solid curve) and for aromatics across from anything other than Gly (green solid curve) in this specific motif are within ± 1 standard deviation (black horizontal line segment) of the general distribution for aromatics in the Top5200 (black dotted curve).

References

1. Richardson JS, Richardson DC (1988) Amino acid preferences for specific locations at the ends of alpha helices. Science 240: 1648-1652.

2. Kapp GT, Richardson JS, Oas TG (2004) Kinetic role of helix caps in protein folding is context-dependent. Biochemistry 43: 3814-3823.

3. Georgiev I, Keedy D, Richardson JS, Richardson DC, Donald BR (2008) Algorithm for backrub motions in protein design. Bioinformatics 24: i196-204.

4. Lovell SC, Davis IW, Arendall WB, 3rd, de Bakker PI, Word JM, et al. (2003) Structure validation by Calpha geometry: phi,psi and Cbeta deviation. Proteins 50: 437-450.

5. Davis IW, Arendall WB, 3rd, Richardson DC, Richardson JS (2006) The backrub motion: how protein backbone shrugs when a sidechain dances. Structure 14: 265-274.

6. Silverman BW (1986) Density Estimation. London: Chapman and Hall.

7. R Development Core Team (2011) R: A Language and Environment for Statistical Computing. Vienna, Austria: R Foundation for Statistical Computing. ISBN 3-900051-07-0. http://www.R-project.org.

8. Cornell WD, Cieplak P, Bayly CI, Gould IR, Merz KM, et al. (1995) A second generation force field for the simulation of proteins, nucleic acids, and organic molecules. J Am Chem Soc 117: 5179-5197.
